# Fruit-Derived Polysaccharides and Terpenoids: Recent Update on the Gastroprotective Effects and Mechanisms

**DOI:** 10.3389/fphar.2018.00569

**Published:** 2018-06-22

**Authors:** Mohammed Safwan Ali Khan, Syeda Umme Kulsoom Khundmiri, Syeda Rukhaiya Khundmiri, Mohammad M. Al-Sanea, Pooi Ling Mok

**Affiliations:** ^1^Department of Pharmacology, College of Pharmacy, Jouf University, Sakaka, Saudi Arabia; ^2^Department of Pharmacology, Anwarul Uloom College of Pharmacy, Jawaharlal Nehru Technological University – Hyderabad (JNTUH), Hyderabad, India; ^3^Department of Biomedical Science, Faculty of Medicine and Health Sciences, Universiti Putra Malaysia, Seri Kembangan, Malaysia; ^4^Department of Pharmaceutical Chemistry, College of Pharmacy, Jouf University, Sakaka, Saudi Arabia; ^5^Genetics and Regenerative Medicine Research Centre, Universiti Putra Malaysia, Seri Kembangan, Malaysia; ^6^Department of Clinical Laboratory Sciences, College of Applied Medical Sciences, Jouf University, Sakaka, Saudi Arabia

**Keywords:** gastric ulcer, anti-*Helicobacter pylori*, polysaccharides, terpenoids, prebiotic, stem cells

## Abstract

Ulceration in the stomach develops in peptic ulcer disease when there is a loss of protective mucosal layers, particularly in *Helicobacter pylori* infection. Antibiotic therapy has failed to eradicate and impede the colonization of *H. pylori*. Despite given treatment, recurrent bleeding can occur and lead to death in the affected individual. The disease progression is also related to the non-steroidal inflammatory drug and stress. There are extensive research efforts to identify the gastroprotective property from various alkaloids, flavonoids, and tannins compounds from plants and marine. These natural products are believed to be safe for consumption. However, not much attention was given to summarize the carbohydrate and terpenoidal anti-ulcer compounds. Hence, this review will cover the possible mechanisms and information about acidic hydroxylans, arabinogalactan and rhamnogalacturon; and limonene, pinene, lupeol, citral, ursolic acid and nomilin to exemplify on the gastroprotective properties of polysaccharides and terpenoid, respectively, obtained from fruits. These compounds could act as a prebiotic to prevent the inhabitation of *H. pylori*, modulate the inflammation, suppress gastric cancer growth, and capable of stimulating the reparative mechanisms on the affected regions. Finally, this review provides the future research prospects of these natural compounds in an effort to develop new therapy for gastrointestinal tissue healing.

## Introduction

Pepticulcer disease (PUD) is a complication that arises due to the imbalance between offensive agents (e.g., acid and pepsin), and protective factors (e.g., mucin, bicarbonate, prostaglandin, nitric oxide, and growth factors) in the gastro-intestinal tract (GIT; Niv and Boltin, 2012; Prabhu and Shivani, 2014; Taş et al., 2015). PUD affects an average of four million people annually (Thorsen et al., 2013), with a higher mortality rate recorded in patients suffering from gastric and duodenal ulcers (Thorsen et al., 2013; Zhang et al., 2016). Infection of *Helicobacter pylori* (*H. pylori*), a type of gram negative bacteria, in the gastrointestinal tract significantly increases the risk of ulceration, bleeding, and ultimately death in the untreated individual (Guariso and Gasparetto, 2012).

The pathogenesis of *H. pylor*i in the development of ulcer has been described in detail by Kao et al. (2016). The invading *H. pylori* could anchor through the binding of adhesin and receptors on the epithelial layer and survive in the acidic milieu in the stomach. Released bacterial toxins such as cytotoxin-associated gene A (cagA) and vacuolating cytotoxin A (vacA) were found to affect cellular proliferation, trigger inflammation, gastric atrophy, and lead to cancer development (Kao et al., 2016).

In addition, continuous stress and long-term medication of non-steroidal anti-inflammatory drugs (NSAID), for example, aspirin for hypertensive patients, are adding to the risk for hospitalization too (Sekhar et al., 2011; Chung and Shelat, 2017). Stress worsens gastric ulcer in patients as it could induce high secretion of histamine and acid-pepsin, reduce mucous production and affects the gastric motility, and weakens the immune defense (Song et al., 2013). NSAID, meanwhile, could damage GIT by decreasing the synthesis of various prostaglandin, (Drini, 2017) disrupting the mucosal protective (Niv and Boltin, 2012; Schellack, 2012), and interferes with the mucosal blood flow which is essential for healing (Kwiecień et al., 2015; Bjarnason et al., 2018). New insights have proposed that these risk factors might modify risk of recurrent bleeding in patients previously infected with *H. pylori* (Chan et al., 2013; Sostres et al., 2014).

Current therapeutic strategy for PUD has focused mainly on reducing gastric acidity and strengthening the gastric mucosal barrier from further physical and chemical insults (Fashner and Gitu, 2015). The various types of drug differ from being a H_2_ receptor antagonist (cimetidine and ranitidine), proton pump inhibitor (omeprazole and lansoprazole), or cytoprotective agents (sucralfate or prostaglandin analogs) (Awaad et al., 2013). To eliminate *H. pylori*, clinicians have resolved to the use of antibiotics such as amoxicillin or clarithromycin, with little success (Awaad et al., 2013). Many of these drugs confer undesirable subsequent health complications including hematopoietic disorders, hypersensitivity (Jalilzadeh-Amin et al., 2015), arrhythmias (Iyer and Alexander, 2016), and gynaecomastia (Goldman, 2010).

**FIGURE 1:**
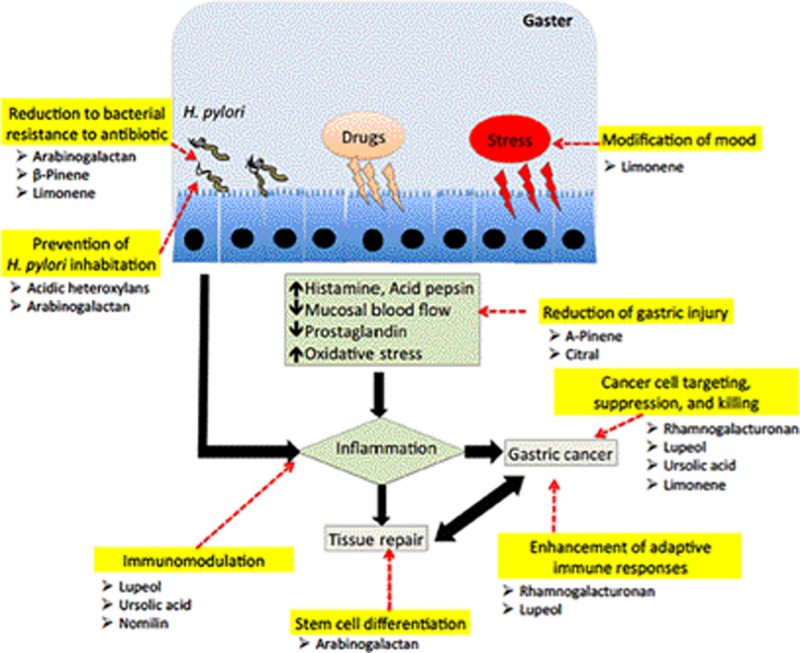
The proposed mechanisms of action of polysaccharides and terpenoids in fighting against disease progression of gastric ulcer due to infection of *H. pylori*, NSAID and stress factors. These compounds could act as a prebiotic, reduce bacterial resistance to antibiotic treatment, reduce gastric injury, modulate the immune responses, stimulate stem cell proliferation and differentiation, and capable of targeting and killing gastric cancer cells.

Alternative treatment using plant compounds such as alkaloids, flavonoids, and tannins, and marine compounds, have been discovered to be useful to reduce severity of ulceration (Khan, 2011; Khan et al., 2011; Ahmed et al., 2013; Ali Khan et al., 2013, 2017). These extracts may offer effective, cheap, and easily available form of treatment for many individuals afflicted with PUD and dyspepsia worldwide (Singh et al., 2008). However, the information on the gastroprotective effect of the polysaccharide and terpenoid classes in the plants, particularly in fruits, are greatly lacking. Hence, we review and discuss the gastroprotective properties of these two compounds and hypothesize on the possible mechanisms of action (**Figure 1**).

## Polysaccharides

### Acidic Heteroxylans

A hemicellulose, acidic heteroxylan is present in *Olea europaea* fruit (Xyl2GlcA2) (Reis et al., 2004). Similar xylans extracted from *Maytenus* *ilicifolia* Mart. Ex Reissek leaves of Celastraceae, and *Phyllanthus niruri* L. from Phyllanthaceae exhibited anti-ulcer activity (Yamada, 1994). Earlier researchers have attributed the anti-ulcer activity of these hydroxylans to their gastric acid inhibitory property (Nergard et al., 2005), and reported that it could form a protective layer on the mucosal surface and increase the production of mucous (Matsumoto et al., 1993).

A significant benefit of acidic heteroxylans has been the ensuing findings that xylooligosaccharide, from which it is extracted, possesses prebiotic property (Aachary and Prapulla, 2011). According to Valcheva and Dieleman (2016), dietary prebiotics are defined as “selectively fermented ingredients that result in specific changes in the composition and/or activity of the gastrointestinal microbiota, thus conferring benefit(s) to human health.” Before this, Homan and Orel (2015) suggested to add probiotics in the classical triple therapy which consists of a proton pump inhibitor and a double antibiotic treatment in the standard regiment to eradicate *H. pylori*. Supplementation of probiotics, such as *Lactobacillus* and *Bifidobacterium* species, showed significant improvement in *H. pylori* eradication program in adults and pediatrics (Ojetti et al., 2012; Zheng et al., 2013; Li et al., 2014), and significantly reduced the side effects of classical triple therapy (Ruggiero, 2014). Nevertheless, when prebiotic was given in combination with probiotics and classical triple therapy to the patients in a clinical trial in 2016, the results indicated a significantly improved eradication rate of *H. pylori* compared to the control armed group (Shafaghi et al., 2016). Hence, more studies should be conducted to asses the potential of xylans to confer protection against *H. pylori* infection. Similarly to some plant-derived polysaccharides, it may prevent initial docking of the bacterium to the gaster cell wall and inhibit cell colonization by blocking the specific carbohydrate receptors involved in host–bacteria interactions (Wittschier et al., 2009; Menchicchi et al., 2015; Chandrashekar and Dharmesh, 2016).

### Arabinogalactan

Arabinogalactan, a polysaccharide is found in the cell wall of higher plants (Fincher et al., 1983). It is also a component of gums and exudates (Fincher et al., 1983; Delgobo et al., 1998; Menestrina et al., 1998). Arabinogalactan is also found in fruits such as mango (*Mangifera indica* L.) (Tamiello et al., 2018), *Lycium ruthenicum* Murr. (Peng et al., 2012), and edible jambo (Tamiello et al., 2018). Earlier researches in 2000s demonstrated that the arabinogalactan could protect gastric tissue by reducing acid and pepsin secretion (Yamada, 1994), enhancing mucous synthesis (Matsumoto et al., 1993), and by coating the mucosal membranes (Nergard et al., 2005).

In another report, arabinogalactan protein JC from *Jatropha curcas* seed endosperm was shown to demonstrate ability to induce cellular differentiation via stimulation of transforming growth factor-beta (TGF-β) (Zippel et al., 2010). In patients who demonstrated well-healing gastric uclers, TGF-β and its receptors were highly expressed in gastric mucosa (Shih et al., 2005). It is possible that arabinogalactan could induce the gastric stem cells to renew and differentiate into functional mucosal layer through TGF-β signaling pathway (Sakaki-Yumoto et al., 2013). Cellular differentiation into parietal, chief, pit, and hormone-secreting enteroendocrine cells are believed to derive from the gastric stem cells, located in the isthmus of gland units (Mills and Shivdasani, 2011; Kim and Shivdasani, 2016). There is a suggestion that even after visual evidence of healing after ulceration, the newly generated epithelium are aberrant and increase the susceptibility to *H. pylori* infection (Aihara et al., 2016). Therefore, the consumption of arabinogalactans in the affected patients may be helpful to resist possible infection by enhancing the body immunity.

Ample studies have investigated on the different possible responses of immune stimulation by arabinogalactans (Udani, 2013). The long chain-specific arabinogalactan can pass through GIT and activate the gut-associated lymphoid tissue. It is possible that this polysaccharide mimics the capsular antigen of potentially pathogenic encapsulated bacteria, and capable of priming the body to produce sufficient immunity to defend against invasion of comparable pathogens (Udani, 2013). There are sufficient evidences showing that larch arabinogalactans can stimulate the innate immune activity of natural killer cells and macrophages, and secretion of pro-inflammatory cytokines in cell and animal models (Dion et al., 2016). Meanwhile, Udani et al. (2010) demonstrated that arabinogalactans could induce greater antibody response against inactivated pneumococcal antigen in adult human subjects compared to control group treated with placebo. In addition, the high expression of TGF-β could inhibit the expression of inducible NO synthases (iNOS) in mesenchymal stem cells (MSCs) (Xu et al., 2014). MSCs is a type of adult stem cells of mesodermal origin, which were found to be present in the gastric submucosal layer (Kim et al., 2013). Inhibition of iNOS will stimulate the pro-inflammatory activity of MSCs, leading to an enhanced adaptive immunity (Mok et al., 2013). Hence, there is a great potential to use arabinogalactans as a supplement to prevent or clear *H. pylori* infection by strengthening the body immunity.

### Rhamnogalacturonan

The rhamnogalacturonan (RG) is a polysaccharide which is present in the grapes (Pabst et al., 2013), ginseng (Yu et al., 2010), and leaves of the plant *Acmella oleracea* (L.) (Asteraceae) known as jambu by the natives (Nascimento et al., 2013). It has a molecular weight of 226 kDa and shows the presence of uronic acid, galactose, arabinose, rhamnose, and glucose (Nascimento et al., 2013). The anti-ulcer activity of RG from *Acmella oleracea* (L.) (Asteraceae) has been studied by using ethanol induced gastric lesions in the Wistar rats. At a dose of 1.5 mg/kg, RG was shown to effectively confer gastroprotective activity in the rats, in comparison to the use of standard drug of Omeprazole at high dose of 40 mg/kg (Nascimento et al., 2013). It is possible that the anti-ulcer activity of RG may act by decreasing the secretion of acid and pepsin, and increasing the mucous production, hence forming a protective coating on the stomach epithelial layer (Yamada, 1994; Nergard et al., 2005).

To date, whether or not RG will have potential significance in treating gastric cancer is not well described. However, RG seems capable of eliciting sufficient immune responses to eradicate tumor cells. For instance, in a study conducted by Park et al. (2013), a ramified form of RG (RG-2) extracts from *Panax ginseng* were able to enhance antigen presentation and stimulatory capability of dendritic cells to CD8+ T-lymphocytes after priming with ovalbumin-expressing EL-4 (EG7) tumor cells. Further findings from the *in vivo* study using transgenic mouse model supported the notion that the RG could have activated DC through *Toll like receptor 4*, resulting in phenotypic maturation of DC and activation of mitogen-activated protein kinase (MAPK) signal pathway. The study also demonstrated successful targeting and ultimately, inhibition of EG7 lymphoma cell growth (Park et al., 2013). Therefore, it is worthy for us to explore into the feasibility to use RG as a natural drug product to prevent gastric cancer formation as a result of *H. pylori* infection, and study the possible mechanisms involved in the suppression of gastric cancer cells before advancement to later stages.

## Terpenoids

Terpenoids are present as secondary metabolites along with other components of alkaloids and flavonoids in plants. Classifications of these metabolites are based on the number of basic unit known as isoprenoid, which is a five-carbon isoprene (C_5_H_8_). The classes include monoterpenes (C10), sesquiterpenes (C15), diterpenes (C20), and triterpenes (C30) (Kuttan et al., 2011). Being an ubiquitous components of our diet, many studies have been conducted to determine the various biological activities of terpenoids and identify the beneficial effects it has on human health (Jia et al., 2013; Cho et al., 2017). Here, we will update mainly on the gastroprotective role of some subclasses of fruit monoterpenoids, triterpenoids, and pentacyclic terpenoids.

### Monoterpenoids (Limonene, Pinene, and Citral)

The essential oil of *Citrus lemon* (L.) Burm. f. (CL) fruit bark contains cyclic monoterpenoids, limonene (LIM), and beta-pinene (β-PIN), mostly (Rozza et al., 2011). LIM is also present in the essential oil extracted from *Citrus aurantium* L. (OEC) (Rutaceae) (Sun, 2007). Meanwhile, citral is an acyclic monoterpenoid, a naturally occuring β-substituted vinyl aldehyde, found in lemons, limes, and oranges (Oliveira et al., 2014). Other monoterpenoids such as α-pinene (α-PIN), β-myrcene, δ-3-carene, terpinolene, and camphene can be isolated from *Cupressus arizonica* Greene fruit oil (Hassanpouraghdam, 2011).

Monoterpenoids are believed to be capable of modifying the harmful effect of stress and NSAID on gastric injury. In an *in vivo* study, oral treatment with D-LIM was found to alter the mood of a group of animal undergoing non-pathological stress stimulating anxiety, in which they developed a more ludic activity (d’Alessio et al., 2012). LIM could neutralize stomach acid and enhance peristaltic movements, and was indicated for the treatment of heartburn and gastro-intestinal reflux disorder (Wilkins, 2002; Moraes et al., 2009; Patrick, 2011). Meanwhile, α-PIN exhibits a significant inhibition of gastric mucosal lesions induced by ethanol, which might be associated, at least in part, with an increase of mucus secretion and reduction of gastric hydrogen ion secretion (Pinheiro Mde et al., 2015). Long term usage of NSAID such as naproxen could cause gastric ulcer. Ortiz et al. (2010) demonstrated that delivery of citral, or a combination of citral and naproxen, in addition to having an additive anti-nociceptive effect, could counter the induction to gastric injury. The authors suggested that citral and naproxen could have interacted at systemic levels, hence, citral might be useful to be prescribed together to patients taking NSAID to reduce its side effects (Oliveira et al., 2014).

LIM and β-PIN possess microbial activity to fight against infection from virus, for instance, herpes simplex viruses, rhinoviruses, and infectious bronchitis viruses (Astani and Schnitzler, 2014). Due to the proven anti-microbial activities of the essential oils, it has been suggested that these oils might be useful to reduce the bacterial resistance, particularly in *H. pylori* infection (Chouhan et al., 2017). The decrease of eradication rate of *H. pylori* are attributed to an increase in the antibiotic resistance rates, worldwide (Ghotaslou et al., 2015). In an *in vitro* study, the *Citrus lemon* and LIM have demonstrated anti-*H. pylori* activity with a minimum inhibitory concentration (MIC) value of 125 and 75 μg/mL, respectively (Rozza et al., 2011). Meanwhile, β-PIN is less effective in completely inhibiting the growth of *H. pylori* with a MIC of 500 μg/mL (Rozza et al., 2011). It is interesting to note that β-PIN is also efficient in promoting the survival of probiotics bacteria, and proven to positively suppress the growth of pathogenic microorganisms in the dairy products (Mahmoudi et al., 2017). Whether or not a combination of β-PIN and probiotics could be used as an adjuvant therapy with the classical triple therapy to increase the success rate of *H. pylori* eradication will require further testing in the near future. Other anti-microbial mechanisms include the possibility that terpenes could perforate the membrane of Gram-positive and negative bacterial cells, causing leakage of intracellular materials which ultimately leads autolysis (Zengin and Baysal, 2014). This finding might be helpful to contribute to the development of essentials oils from fruits as an alternative to antibiotic treatment, to prevent further progression to gastric ulcer.

Other significant anti-neoplastic evidences of monoterpenoids have been collected from various studies in lymphoma, mammary, lung, and prostate cancer models (Sekhar et al., 2016). In relevance to GIT, the chemopreventive activity of LIM against gastric cancer (Uedo et al., 1999), liver cancer (Sun, 2007), and colorectal cancer (Vigushin et al., 1998) have been documented as early as in the 1990s. Several anti-tumor mechanisms of D-LIM have been identified contributing to this observation. Inhibition of tumor proliferation is mediated through augmentation of Bcl-2-associated X protein (Bax) and B cell lymphoma-2 (Bcl-2) levels and inactivation of Akt signaling. Other than that, cell apoptosis could occur due to the rise of activities in the caspase-dependent mitochondrial death pathways, poly(ADP-ribose) polymerase cleavage and tumor protein 53 (p53) within the cancerous cells (Kapoor, 2013; Zhang et al., 2014). It also possible that D-LIM could reduce oxidative stress and inhibit Ras signaling pathway in response to cancer induction and promotion (Chaudhary et al., 2012). Accompanying these changes are attenuation of tumoral angiogenesis and migration, via down-regulation of vascular endothelial growth factor (VEGF) and matrix metallopeptidase-9 (Kapoor, 2013; Zhang et al., 2014). Meanwhile, α-PIN could initiate cell cycle arrest in gastric cancer SGC-7901 cell line by inducing Ataxia Telangiectasia-Mutated Kinase signaling pathway in response to DNA damage. Following that, tumor suppressor p53 and p21 genes will be activated to allow full execution of its anti-tumor effects (Zhu et al., 2015).

### Lupeol

Lupeol [lup-20(29)-en-3H-ol] is a type of pentacyclic terpenoid, which can be extracted from olives (3 μg/g), mangoes (1.80 μg per pulp), and Japanese pear (175 μg/g of twig bark) (Nakamura and Watanabe, 2017). Previous studies showed that this compound is involved in lipid metabolisms, and possesses anti-protozoal and anti-microbial activities (Iman et al., 2007; Martelanc et al., 2007; Prasad et al., 2007; Wal et al., 2015).

Generally, lupeol shows gastroprotective activity against ulcerogenic effects of ethanol in the experimental animals (Lira et al., 2009). In the case of *H. pylori* infection, neutrophils release large quantities of microbicidal superoxide anion during respiratory burst. This condition exacerbates inflammation in the gastric mucosa (Bonacorsi et al., 2013). Gallo Margareth and Sarachine Miranda (2009) have extensively detailed the anti-oxidant and anti-inflammatory mechanisms of lupeol extracted from different region of plants. However, changes in the lupeol molecular structure will respond differently to various inflammatory conditions. For instance, in a topical treatment, lupeol and its hemisuccinyl ester would enhance epidermal tissue reconstitution, while acetylation and palmitoylation of the OH-3 group would reduce it (Gallo Margareth and Sarachine Miranda, 2009). Hence, it is worthy for us to explore on the potential use of various lupeol compounds from fruit extracts to reduce oxidative stress and inflammation in PUD affected by *H. pylori*. Also, it may be useful to prevent gastric cancer development (Saleem et al., 2004; Murtaza et al., 2009) as lupeol could downregulate signal transducer and activator of transcription 3 (STAT3) signaling pathways in tumor cells (Siveen et al., 2014). Another *in vitro* study demonstrated that lupeol could inhibit growth of gastric cancer cell lines, by indirectly stimulating the proliferation and cytotoxic activity of natural killer cells (Wu et al., 2013).

### Triterpenoids (Ursolic Acid and Nomilin)

Ursolic acid is found as secondary metabolite, in the form of pentacyclic triterpenoids, in apple (*Malus domestica*) fruit peel (Woźniak et al., 2015; Lv, 2016), cherries (*Cornus officinalis)* (Liu, 1995; Chattopadhyay et al., 2002; Woźniak et al., 2015), and berries (Kondo et al., 2011; Woźniak et al., 2015). With regards to gastric cancer, ursolic acid have been tested in a few cell lines (AGS, BGC823, SGC7901, and SNU-484) and were shown to induce apoptosis via downregulation of Bcl-2 and Bax level, inhibition of cyclooxygenase 2, and increase in caspase-3, and -8 activities (Liu, 1995; Chattopadhyay et al., 2002; Woźniak et al., 2015). Few researches have encapsulated ursolic acid in liposomes and reported an enhanced efficiency in targeting and eliminating cancer including the remnant cancer stem cells (Wang et al., 2016; Ying et al., 2017). Pre-treatment of BGC-823 human adenocarcinoma gastric cancer cell line with ursolic acid could sensitize the cells to the toxicity of radiation (Yang et al., 2015). Meanwhile, nomilin is a form of oxidized triterpenes which give the bitterness in the orange peel (Raphael and Kuttan, 2003). Both nomilin and UA could stimulate the proliferation and differentiation of hematopoietic stem cells in the bone marrow to give rise to more immune cells, thus, enhancing the innate and adaptive immunity. These triterpenoids are also capable of modulating immune response and inhibited delayed type of hypersensitivity reaction (Raphael and Kuttan, 2003).

## Future Prospects of Polysaccharides and Terpenoids for Pud

Natural products have been used traditionally and considered relatively gentle to the body compared to synthetic drugs (Kangwan et al., 2014). Collectively, current review shows that the polysaccharide and terpenoid compounds possess preventive and curative properties for PUD, acting through wide mechanisms. More studies should be conducted to affirm these in fruit extracts, and determine the optimal effective dosage when used alone as probiotic supplement, or in combination with other drugs or phytochemical mixtures to clear *H. pylori* infection and ultimately, induce healing of PUD. The use of phytochemical mixtures, at an optimal amount, have been reported to render a synergistic effect in treating chronic diseases including cancers (Breda and Kok, 2017). Despite that, no other investigations have been carried out to determine the effect when two or more types of polysaccharides or terpenoids are combined in treating diseases, except the demonstration that a mixture of blueberry and apple juices could confer increased protection against *ex vivo* induced hydrogen peroxide (H_2_O_2_) oxidative DNA damage in 168 healthy volunteers (Breda and Kok, 2017).

**FIGURE 2:**
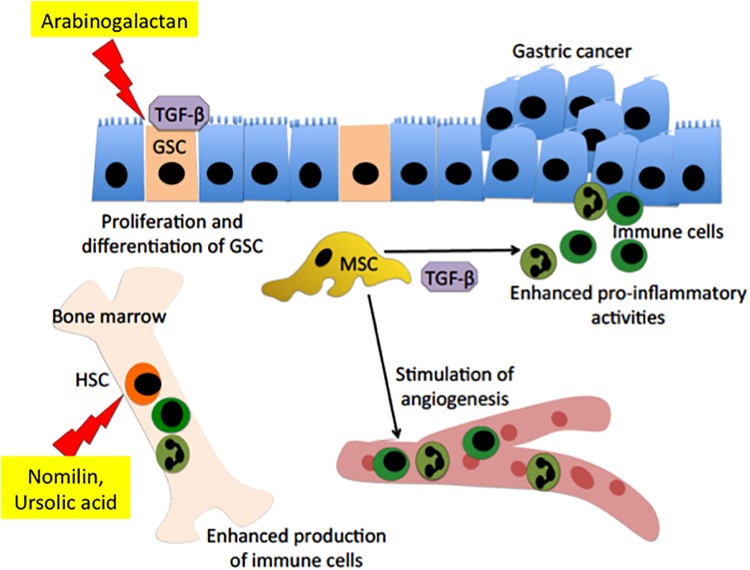
Interaction between fruit-derived polysaccharide or terpenoids and stem cells in gastric mucosal tissue repair and prevention of gastric cancer growth. Arabinogalactan is capable of stimulating expression of transforming growth factor (TGF-β) and its receptor, which will in turn induce proliferation and differentiation of gastric stem cell (GSC), thereby inducing rapid tissue healing. The high level of TGF-β will inhibit the expression of inducible NO synthases (iNOS) in mesenchymal stem cell (MSC). Inhibition of iNOS will stimulate the pro-inflammatory activity of MSCs, leading to an enhanced adaptive immunity. Meanwhile, nomilin and ursolic acid could stimulate the hematopoietic stem cell (HSC) in the bone marrow to produce more immune cells. The increase in the immune cells could kill maltransformed cells and therefore suppress gastric cancer growth.

These phytochemicals may also potentially stimulate the development of new research area, particularly in regenerative medicine, to study the interactions between these compounds and normal resident or distal stem cells to trigger tissue healing in GIT (**Figure 2**). Much is not known with regards to how these compounds could effect or awake the function of MSCs in the gastric submucosal layer. A report has shown that the transplantation of these cells could reduce gastric ulceration and improve healing through the secretion of VEGF (Wang et al., 2015). Of note, generation of human gastrointestinal organoid from pluripotent stem cells (Dedhia et al., 2016) and epithelial stem cells have been reported (Nakamura and Watanabe, 2017). The use of these compounds may aid in directing the differentiation pathways of these stem cells to functional gastric cells. In addition to that, being that oral route is an attractive choice for drug administration, polysaccharides such as RG has been used to make microcapsules for targeted delivery in the GIT. Polysaccharides are non-toxic, possess good biocompatibility, and showed capability to release therapeutic drug in a controlled manner (Svagan et al., 2016). Hence, it is feasible to use RG as a vehicle to deliver drug for more efficient treatment for PUD. Also, there is a potentially bright prospect to use these compounds as anti-cancer agent.

Despite evidences pointing to the beneficial effect on human health, there is a need to determine the toxicological profile and off-target effect before application. For example, high doses range between 40 and 50 μM α-PIN have been identified to interfere with mitotic process, causing genomic instability in non-cancerous mammalian cells (Catanzaro et al., 2012). Meanwhile, in a clinical study conducted in China (ChiCTR-ONC-12002385), intravenous injection of ursolic acid encapsulated in liposomes into healthy adult volunteers produced manageable toxicities with a maximum tolerated dose of 98 mg/m^2^. At high doses (74, 98, and 130 mg/m^2^), the subjects experienced hepatotoxicity and diarrhea (Wang et al., 2013).

## Conclusion

In summary, the present paper reveals the significance of polysaccharides (acidic heteroxylans, arabinogalactan, and RG) and terpenoids (limonene, pinene, citral, lupeol, ursolic acid, and nomilin) as potential gastroprotective agents from insult of *H. pylori* infection, NSAID and stress factors. These compounds may as prebiotic or interact with gut microbiota to impede the adherence, colonization, and invasion of *H. pylori* into the gastric cell wall. In addition to that, it can stimulate the resident stem cells to initiate mucosal cell proliferation and differentiation, and synergistically modulate the immune responses in the affected region. These compounds could also prevent gastric cancer formation, and suppress cancer growth, which is prevalent in *H. pylori* infected patients. Despite the reported hypothesized mechanisms, further exhaustive mechanistic and toxicological studies are necessary to transform these lead compounds into commercial drugs in the near future.

## Author Contributions

MSAK and PLM designed this work and figures, collected and analyzed the data, co-wrote the manuscript, and edited the manuscript. SUKK, SK, and MA-S gathered the data.

## Conflict of Interest Statement

The authors declare that the research was conducted in the absence of any commercial or financial relationships that could be construed as a potential conflict of interest.
